# Protection of Workers Exposed to Radiofrequency Electromagnetic Fields: A Perspective on Open Questions in the Context of the New ICNIRP 2020 Guidelines

**DOI:** 10.3389/fpubh.2022.875946

**Published:** 2022-06-02

**Authors:** Peter Jeschke, Carsten Alteköster, Kjell Hansson Mild, Michel Israel, Mihaela Ivanova, Klaus Schiessl, Tsvetelina Shalamanova, Florian Soyka, Rianne Stam, Jonna Wilén

**Affiliations:** ^1^Federal Institute for Occupational Safety and Health, Dortmund, Germany; ^2^Institute for Occupational Safety and Health of the German Social Accident Insurance, Sankt Augustin, Germany; ^3^Department of Radiation Sciences, Radiation Physics, Umeå University, Umeå, Sweden; ^4^National Centre of Public Health and Analyses, Sofia, Bulgaria; ^5^Austrian Workers' Compensation Board, Vienna, Austria; ^6^National Institute for Public Health and the Environment, Bilthoven, Netherlands

**Keywords:** occupational exposure, reduction factors, electric field, magnetic field, EMF Directive 2013/35/EU, uncertainty, ICNIRP 2020 RF Guidelines

## Abstract

Workers in occupational settings are usually exposed to numerous sources of electromagnetic fields (EMF) and to different physical agents. Risk assessment for industrial workplaces concerning EMF is not only relevant to operators of devices or machinery emitting EMF, but also to support-workers, bystanders, service and maintenance personnel, and even visitors. Radiofrequency EMF guidelines published in 2020 by the International Commission on Non-Ionizing Radiation Protection (ICNIRP) may also be indirectly applied to assess risks emerging from EMF sources at workplaces by technical standards or legislation. To review the applicability and adequacy to assess exposure to EMF in occupational settings in the European Union, the most current ICNIRP guidelines on radiofrequency EMF are reviewed. Relevant ICNIRP fundamentals and principles are introduced, followed by practical aspects of exposure assessment. To conclude, open questions are formulated pointing out gaps between the guidelines' principles and occupational practice, such as the impact of hot and humid environments and physical activity or controversies around ICNIRPS's reduction factors in view of assessment uncertainty in general. Thus, the article aims to provide scientific policy advisors, labor inspectors, or experts developing standards with a profound understanding about ICNIRP guidelines' applicability to assess hazards related to radiofrequency EMF in occupational settings.

## Introduction

In recent years, both the International Commission on Non-Ionizing Radiation Protection (ICNIRP) and the Institute of Electrical and Electronics Engineers (IEEE) have published a new set of guidelines (1, 2) addressing the safety and health of workers and the general public when exposed to electromagnetic fields (EMF). Both guidelines are internationally well-acknowledged among regulators and standardization bodies. Especially for the European Union and its Single Market, occupational and general public legislation or recommendations as well as (product) standards were founded on several ICNIRP guidelines in the past. Within the framework Directive for safety and health of workers at work 89/391/EEC (3), the 20th individual Directive regarding the exposure of workers to EMF 2013/35/EU (4) builds upon ICNIRP (5) regarding static magnetic fields (6) regarding low frequency EMF, and (7) regarding radiofrequency EMF (RF EMF). Two “non-binding” guides' published by the European Commission provide a categorization of different working environments and strategies for exposure assessment, risk reduction measures and workers at particular risk, as well as a collection of case studies (8, 9).

Currently, the situation in the European Union legal area becomes challenging, as existing regulatory limits relate to ICNIRP's 1998 and 2010 guidelines (6, 7), whereas updates of some technical standards already refer to ICNIRP's 2020 guidelines (1), especially those applicable to the Frankfurt-Agreement^1^ (10), e.g., IEC 62232 Ed.3 (11), IEC 62493 Amd. 1 Ed. 2 (12), or IEC TR 63377 Ed. 1 (13). To avoid such a set of two different reference systems, currently the Scientific Committee on Health, Environmental and Emerging Risks (SCHEER) is evaluating the need for an update (14) of the EMF-Directive (4) to accommodate the newly introduced safety and health guidelines by ICNIRP (1). If the update process will be initiated to apply (1) in updated European legislation, it will follow the tradition of previous guidelines dating back as far as 1998 (7).

ICNIRP's RF guidelines (1) derive, motivate, and define a revised safety concept based on a set of Basic Restrictions (BR) and Reference Levels (RL) to maintain safe exposure to RF EMF. The authors of the present review acknowledge and highly value ICNIRP's scientific approach. We acknowledge, that ICNIRP does not intend to provide a blueprint for legislation, nor for technical standards. Respectively, ICNIRP (1) does not always consider practical application of its safety concept. To apply practical experience to ICNIRP's 2020 guidelines (1), the present review, respectively, aims to:

- derive open questions when applying (1) in occupational safety and health (OSH) risk assessment practice and
- enable an efficient and effective OSH practice by proposing solutions for both risk assessment based on workplace measurements and computational exposure determination (e.g., addressing accuracy vs. practicability).

The following contribution comprises only occupational exposure (meaning employment related exposure settings) associated with established acute thermal effects, leaving EMF exposure of the general public and research into non-thermal effects out of its scope. For various reasons occupational exposure settings differ from exposure settings for the general public; please refer to ICNIRP's 2020 guidelines [(1), p. 484–5]. Two major differences can be highlighted: firstly, the permissible exposure level is elevated by factor 5 compared to general public with a maximum exposure duration of working hours over decades. Secondly many *in vivo* health effect studies are conducted at frequencies relevant for sources of general public exposure (e.g., mobile phones) and exposure levels equal to or lower than the ICNIRP limits for the general public and then extrapolated to occupational exposures. Important intense occupational exposure situations at ISM frequencies, such as 13.56 and 27.2 MHz (or below), are indeed quite rare.

To start with, the most relevant changes in ICNIRP 2020 (1) are summarized, followed by the identification of key challenges for its application in OSH risk assessment practice in chapters 2–4. Assuming the application of International Commission for Non-ionizing Radiation Protection (1) in OSH risk assessment and measurement practice and hence future (European) legislation, the contribution concludes with a summary of open questions which need to be addressed in order to promote efficient and effective OSH risk assessment practice as of chapter 5.

## Fundamentals Of ICNIRP RF Guidelines 2020: What is Different For Workplace Assessment?

This section identifies the main changes introduced in ICNIRP 2020 (1) compared to the previous guidelines (7), particularly where these changes lead to improvements or complications in their application to the occupational setting. Some of these changes were also highlighted in summary published by ICNIRP (15), but additional issues were identified by the authors.

### Scope and General Principles

The new ICNIRP guidelines (1) define occupationally-exposed individuals as adults who are exposed under controlled conditions associated with their occupational duties, trained to be aware of potential radiofrequency EMF risks and to employ appropriate harm-mitigation measures, and who have the sensory and behavioral capacity for such awareness and harm-mitigation response. An occupationally-exposed worker must also be subject to an appropriate health and safety program that provides the above information and protection. This definition is stricter than that in ICNIRP 1998 (7), which defined the occupationally-exposed individuals as adults who are generally exposed under known conditions and are trained to be aware of potential risk and to take appropriate precautions. It is important to note that this new definition may not be directly comparable to the legal definition of a worker in EU or national law, to which the ICNIRP exposure limits may be applied via legislation. ICNIRP's opinion in its 2002 “general approach to non-ionizing radiation protection” (16) was that the relevant authorities in each country should decide on whether occupational or general public guideline levels are to be applied, according to existing (national) rules or policies.

The new guidelines (1) do not apply to exposure of patients undergoing medical procedures (as well as their careers and comforters), which rely on medical expertise to weigh potential harm against intended benefits, since such exposures are managed by qualified medical practitioners. However, cosmetic treatments without control by a qualified medical practitioner are covered by ICNIRP 2020 (1). Volunteer research participants are also outside the scope of the guidelines, provided that an institutional ethics committee approves such participation following consideration of potential harms and benefits. Occupationally-exposed individuals working in all these settings (medical, cosmetic and research) are still within the scope of ICNIRP 2020 (1).

In contrast to ICNIRP 1998 (7), the new guidelines (1) specifically define a pregnant woman (worker) as a member of the general public in terms of the basic restrictions, for both whole-body and local exposure and for exposures longer and shorter than 6 min. Recent modeling studies suggest that exposure of the mother at the occupational basic restrictions can expose the fetus above the basic restrictions for the general public. The above principles on categories of exposure are also reflected in the latest version of ICNIRP's principles for non-ionizing radiation protection (17). With regard to pregnant workers, the latter add that the general public limits apply if a female worker has declared that she is pregnant. As with other groups of workers at particular risk, it may not be possible for an employer to identify this without the worker voluntarily disclosing this information. Like (7), but less extensively (1) states that harmful interactions with active or passive implanted medical devices are outside the scope of the guidelines. The interference of radiofrequency EMF with electrical equipment more generally, which can affect health or safety indirectly by causing equipment to malfunction (electromagnetic compatibility) is also declared outside the scope of ICNIRP 2020 (1).

### Basic Restrictions

Aim of the basic restrictions (BR) in ICNIRP 2020 (1) is to limit the rise in body temperature due to exposure by radiofrequency EMF to a maximum

- of 1°C for the core temperature for whole-body exposure
- of 5°C for local exposure of so-called Type-1 tissue (limbs, pinna of the ear, cornea, anterior chamber and iris of the eye, epidermal, dermal, fat, muscle, and bone tissue), or
- of 2°C for local exposure of so-called Type-2 tissue (head, eye, abdomen, back, thorax, and pelvis, excluding those parts defined as Type-1 tissue).

For exposure of 6 min or longer, the basic restrictions in terms of the specific absorption rate (SAR, in W/kg) are identical to those in ICNIRP 1998, but the averaging time has been increased from 6 to 30 min for whole-body exposure, based on more recent scientific research on the time to reach a steady-state temperature. This means that the whole-body average SAR (wbaSAR) can be higher than the basic restrictions for time intervals shorter than 30 min, while the basic restrictions for local exposure will still protect the worker against hazardous local heating. The averaging time for local exposure of head, torso and limbs remains 6 min. New dosimetric insights have also led ICNIRP to increase the basic restriction for frequencies from 6 to 300 GHz in terms of absorbed power density (S_ab_, in W/m^2^) from 50 to 100 W/m^2^, and to decrease the averaging area from 20 to 4 cm^2^. An additional basic restriction is set for frequencies above 30 GHz, where the absorbed power density averaged over 1 cm^2^ is restricted to 200 W/m^2^. These adaptations are important safeguards for exposure due to microwave technologies that use beamforming, such as those applied in massive MIMO systems for 5G telecommunication.

A new set of basic restrictions was introduced in ICNIRP 2020 (1) for local exposure shorter than 6 min, to prevent excessive local heating in the period where heat has not had time to redistribute to other parts of the body. They are particularly important for frequencies higher than 6 GHz, where the energy is mainly absorbed in superficial body areas (skin, eyes). These basic restrictions are highly relevant for pulsed millimeter waves such as those produced by broadband telecommunication or radar signals. The basic restrictions are set in terms of the specific energy absorption (SA, in J/kg) for frequencies of 400 MHz to 6 GHz and in terms of absorbed energy density (U_ab_, in J/m^2^) for frequencies of 6 to 300 GHz. Their value depends on the exposure duration: the shorter the exposure duration, the lower (stricter) the basic restriction. This definition means they can be applied to continuous wave as well as pulsed EMF. The averaging volume and surface are the same as for exposures longer than 6 min (10 g for SA and 4 cm^2^ for U_ab_). An additional basic restriction is set for frequencies above 30 GHz for the absorbed energy density averaged over 1 cm^2^ to protect against harmful focal beam exposure. ICNIRP has stated that the new limits for local exposure shorter than 6 min ensure that new and future technologies using higher RF EMF frequencies, such as 5G, will not result in excessive temperature rise due to brief exposures (15). However, they do add an extra layer of complexity to the limit system which may complicate workplace compliance assessment.

For frequencies of 0.3–6 GHz, the 1998 ICNIRP guidelines (7) contained an additional basic restriction for localized specific energy absorption to prevent “microwave hearing,” the audible sound that can be generated by thermo-elastic expansion of tissue in the head due to sub-millisecond pulses of radiofrequency EMF. ICNIRP has removed this basic restriction in the 2020 guidelines (1), since it represents only a (possibly disturbing) sensory phenomenon with no evidence that it would cause adverse health effects. For frequencies of 100 kHz−10 MHz (7) also contained basic restrictions for the induced current density to prevent harmful electrical stimulation, but these have been replaced by basic restrictions for the induced electric field in ICNIRP's 2010 guidelines (6). It is the latter basic restrictions for electrical stimulation that were applied as exposure limit values in the EMF Directive (4). Examples of working environments in this frequency range are induction heating and electronic article surveillance systems.

### Reference Levels

Reference levels (RL) in ICNIRP 2020 (1) are derived from the basic restrictions in terms of EMF quantities outside the body that are more easily assessed and provide an equivalent level of protection for worst-case exposure scenarios. Reference levels for whole-body exposure are averaged over 30 min (was: 6 min) and over the space that would be occupied by the body. For frequencies of 100 kHz to 2 GHz [was: 300 GHz in ICNIRP 1998 (7)], reference levels are given for incident electric and magnetic field strength. For frequencies of 2–300 GHz, there are reference levels for incident power density. Compared to ICNIRP 1998 (7), their values are increased (less strict) for frequencies between 100 kHz and 30 MHz, due to new dosimetric insights and smaller computational uncertainties. For frequencies of 2 GHz to 300 GHz, the reference levels are still set in terms of incident power density, with values identical to those in ICNIRP 1998 (7).

New compared to ICNIRP 1998 (7) are reference levels for local exposure longer than 6 min (averaged over 6 min), which are related to the basic restrictions for head, torso and limbs. They are given for the incident electric (E-)field strength, magnetic (H-)field strength and power density. Also new are the reference levels for local exposure shorter than 6 min in terms of incident energy density (U_inc_, in J/m^2^), which are derived from the new basic restrictions for exposure shorter than 6 min and frequencies from 400 MHz to 300 GHz. Like the basic restrictions for short exposures, their value depends on the exposure duration: the shorter the exposure duration, the lower (stricter) the reference level.

As in ICNIRP 1998 (7), a separate reference level (100 mA) is given for limb currents to guard against exceeding the SAR basic restriction when radiofrequency current is concentrated in the thinner muscle layers of the limbs and the joints even when the E-field and H-field strength are lower than the reference levels. However, the applicable frequency range has been extended from 10–110 to 0.1–110 MHz, due to new dosimetric insights. In principle, contact currents caused by radiofrequency EMF interacting with a conducting object which is then touched by the worker could also increase the SAR above the basis restrictions. ICNIRP 1998 (7) provided a reference level for these contact currents, but ICNIRP 2020 (1) no longer does so. ICNIRP argued that it is not possible to provide such a reference level due to the need to account for a variety of parameters that cannot be routinely specified in advance, such as the contact area, resistance between body and object and conductivity of the body tissue. Instead (1) provides guidance for training workers' awareness, avoidance and protective measures.

## Description of ICNIRP'S Operational Adverse Health Effects Thresholds and Characterization of Deeper Tissue

Alongside the introduction of Section “Fundamentals of ICNIRP RF Guidelines 2020: What Is Different for Workplace Assessment?” and acknowledging the rationale of ICNIRP 2020 (1), the goal of ICNIRP's BR for RF EMF shall be emphasized once more: Based on thermophysiological insights, the rise of the body core temperature needs to be limited to +1°C when starting from a steady-state temperature. Additionally, the absolute temperature of the tissue must be limited in order to avoid pain and subsequent thermal damage, which cannot be excluded above 41–43°C. Previously, concerns focussed on cataract formation in the eye (7). A conservative limit of 41°C is adopted as a general threshold for a potentially harmful tissue temperature [please see ICNIRP 2020 (1), p. 489]. It must be noted that (1) implicitly regards such a temperature threshold as permanently endurable.

However, absolute tissue temperature can hardly be the direct basis for formulating BR because the local temperature at workplaces is multifactorial, depending on e.g., ambient temperature, workload, clothing or warm objects nearby. It does appear convenient to reduce all thresholds to relative temperature rises attributed to RF EMF exposure. After all, the equivalence between heat generated by RF exposure and metabolic heat production is well-established. But it must be recalled that a formulation of relative temperature rise as Operational Adverse Health Effects Thresholds [OAHET, cf. (1)] only becomes valid on certain conditions, such as the normothermal temperatures of body regions and their tissues. Normothermal conditions relate to about 28°C for naked persons, and to room temperatures of about 24°C for persons with clothing, and exclude relevant metabolic heat production due to physical work.

Today, BR are usually related to OAHET via numerical dosimetry. At the same time, the number of human *in vivo* RF EMF exposure studies is quite limited. This is particularly the case for intense exposures of healthy adults, with intensities near the limits for occupational exposure. For the frequency range below 6 GHz and exposure of almost the whole-body, the original work of Adair (18) represents the main validation of numerical simulation of human thermoregulation. Apart from recording superficial warming via observation of skin temperature and local sweat rates, Adair's work engages mainly in aspects of regulation and reaching adverse levels of body core temperature in deeper tissue. With this cornerstone, and together with physiological data on body core temperature in laboratory tests on animals (19), whole body RF-warming under average environmental conditions has undoubtedly been extensively studied and the corresponding BR are well-established.

However, apart from localized RF-warming in the head due to mobile phone devices, studies on RF exposure at local (e.g., superficial tissue, finger tips) to body region levels (e.g., whole limb) are much rarer and nevertheless far more relevant for occupational exposure settings. The BR below 6 GHz is once more the SAR in terms of peak spatial SAR averaged over 10 g (psSAR10g). It is a “metric for simultaneously protecting both the internal tissues (e.g., brain) and the skin” [rationale of ICNIRP 2020 (1), p. 507)]. This psSAR10g below 6 GHz covers a frequency range accounting for penetration depths of several decimetres at 100 kHz (considered sub-resonance) down to 8.1 mm at 6 GHz (considered as surface absorption) [(1), p. 504]. Deeper tissue in the extremities and the adequacy of the metric are not explicitly discussed. Contextual dosimetric results like heating factors focus on superficial tissue such as skin. However, for occupational exposure settings such as dielectric RF welding, type-1 tissue regions, like deeper lying muscle and bone tissue in the forearm, obviously are very relevant. Application of relatively high values of up to 20 W/kg (psSAR10g) would benefit from further discussion and more detailed provisions by ICNIRP 2020. Presentation of controlled *in vivo* RF-exposure studies concerning extremities at large might be listed as a research gap.

### Limbs and Choice of OAHET for Type-1 Tissue

ICNIRP 2020 (1) treats limbs as separate category, following IEEE (2), which adapted its definition for extremities to match the whole limbs (including thigh and upper arm). ICNIRP 2020 (1) focusses specifically on the nature of tissue and associates limbs with tissue of type-1 only, and consequently with an OAHET of +5°C for the whole limb.

While this choice of OAHET, e.g. for skin seems plausible (temperatures of skin superficial tissue above +36°C are hardly reached and are thus five degrees off the specified threshold of +41°C), the application to deeper tissue within the limbs is less straightforward and not supported by further references. Other literature, for example, speaks (also with respect to deeper tissue) of a peripheral compartment that is only 2–4°C cooler than the body core under normothermal conditions, and retains temperatures similar to that of the body core when being in a warm to hot environment (20).

A more detailed discussion of the literature basis for ICNRIP's choice would be beneficial to clarify the role of the extremities' deeper tissue, which is of particular relevance for occupational exposure. One example for an open question is the classification of peripheral nerve tissue, which is certainly also present in the extremities, as type-1 tissue.

One should additionally note that, according to ICNIRP 2020 (1), the tissues fat, muscle and bone, defined as type-1, are also allowed to reach the OAHET of +5°C when being situated on head & torso. Stricter limits in the head apply apparently only to brain and eye-ball tissue, while for skull and other parts of the head an OAHET of +5°C seems to be tolerated. In particular with occupational BR like psSAR10g = 10 W/kg for the head, those temperature rises in non-brain tissues may almost be reached, please refer to Morimoto et al. (21) and its discussion in Foster et al. (22). Such results call for better justifications by ICNIRP 2020 (1), of both the choice of reduction factors, and of the validity of attributing significantly different OAHET to neighboring tissues of the same body region.

### Choice of Reduction Factors From OAHET to Basic Restrictions Concerning Occupational Limb Exposure

ICNIRP 2020 (1) elaborates on reduction factors between effect threshold (or OAHET) and BR in somewhat more detail than previously. While those factors are undoubtedly substantial for the general public, those for the BR of workers are much smaller. For local (peak spatial) SAR—both on head and torso and for the limbs—reduction factors equal only a value of 2. Hence, for a continuous wave exposure, a RF-induced, permanent temperature rise of +2.5°C^2^ in all of the limbs corresponds to the BR and thus complies with (1) for occupational exposures; implicitly, within the uncertainty budget said to be covered by the reduction factor, ICNIRP deems +5°C as permanently safe. With regards to a conservative derivation of BR such a small reduction factor poses a challenge for accommodating all worst-case exposure scenarios, variety of workers' anatomy, positions, and work conditions (please refer to Sections “Problems With OAHET in Co-Exposure With Harsh Work Environments and Uncertainty of Dosimetric and Thermal Simulations in Basic Restriction Assessment at Frequencies Below 6 GHz”).

Note also that according to ICNIRP 2020 (1), reference levels are derived from basic restrictions without further reduction factors. Consequently, application of reference levels (which is the most common method of assessing a workplace) will not add an extra margin of safety to the narrow reduction factor in the BR. In fact, for the similar relation between whole-body RL and wbaSAR, several exposure setups are meanwhile acknowledged by ICNIRP 2020 (1) where reference levels are actually not conservative enough. For a short discussion on the newly introduced RL for local exposure, see also Sections “Spatial Averaging of Basic Restrictions and Local Reference Levels and Local Reference Levels for Exposure Durations of <6 Min”.

### Problems With OAHET in Co-exposure With Harsh Work Environments

RF EMF exposure results in heating of the human body which in turn can lead to adverse health effects if either local or body core temperatures become too high. Since RF EMF is not the only possible source of body heating in the environment, it is impossible to set EMF limits which prevent a temperature rise above a specified absolute temperature. It is only possible to limit the relative temperature increase. This can make it difficult to provide appropriate occupational limits in situations where other significant heat sources are present as well. In such cases, RF EMF exposure must be considered in conjunction with those other sources. ICNIRP 2020 (1) acknowledges this under risk mitigation considerations for occupational exposure and furthermore points out that: “Similarly, it is also important to consider whether a person has an illness or condition that might affect their capacity to thermoregulate, or whether environmental impediments to heat dissipation might be present” [(1), p. 500]. They propose that workers should have suitable means of monitoring their body core temperature to prevent problems. Looking into IEEE 2019 (2), one finds a similar discussion of the problem with the statement: “The larger issue of worker protection against heat strain under extreme environmental or work conditions is beyond the scope of this standard” [(2), p. 142]. This shows that for the possible application of ICNIRP 2020 (1) in European legislation, it is not sufficient to simply transpose (1) one-to-one to provide a suitable system for worker protection. Especially for RF exposure care must be taken that co-exposure scenarios are sufficiently accounted for.

Unfortunately, ICNIRP's discussion of occupational exposure does not explicitly answer the question to which extent their occupational limits (both BR and RL) are deemed to be unconditionally applicable. Readers of the guidelines that are unfamiliar with the underlying literature of the last decades thus might doubt the applicability of occupational limits outside situations under normothermal conditions and low physical activity. The findings in the RF-exposure studies of Adair (23) on wbaSAR together with the substantial reduction factor for whole-body exposure will make hyperthermia indeed unlikely, even in warm environment. Nevertheless, few studies have tackled the combination of hot environments, clothing and physical exercise. Even Moore et al. (24), being the only exception that could ease concerns, has no legal relevance for occupational risk assessment practice.

In contrast to whole body exposure, occupational exposure of the limbs is investigated in the literature only on rare occasions. ICNIRP's assumption, namely that the limbs' tissue “[…] is unlikely to increase local temperature by more than 2.5°C, and given that limb temperatures are normally below 31–36°C, it is unlikely that RF EMF exposure of limb tissue, in itself, would result in either pain or tissue damage” [(1), p. 501] is not beyond debate [see Section Limbs and Choice of OAHET for Type-1 Tissue and (20)]. Moreover, the statement “in itself” once more points implicitly to other sources of heat, which would have to be assessed together with RF EMF. For practical applicability in OSH risk assessment, this seems somehow problematic.

Finally, (1) correctly points out the importance of health and safety programs and workers' training. However, the framework directive (3) prioritizes collective Technical and Organizational protective measures over Personal (individual) protective measures. An individual's reaction to intense RF-exposure due to thermal discomfort or even pain thus can never be a major item to assure personal safety, especially for penetration of RF EMF in deep tissue with no receptors, such as muscle and bone tissue. Instead, the technical setup must guarantee—with the aid of exposure limits appropriate for the given situation—that exposure remains sufficiently low.

## Practical Aspects of Compliance Assessment

### Reference Levels

In ICNIRP 1998 (7) RL were given for E- and H-fields as RMS (root mean square—quadratic mean over time) values for whole-body exposure of workers in Table 6 and Note 5 additionally specified limits for peak values (100 kHz−300 GHz). Although not explicitly mentioned, it can be assumed that these peak values are not RMS quantities anymore, but rather, as the name suggests, instantaneous peak values. Another possible interpretation would be that the limits still refer to RMS quantities, but that the RMS averaging time is reduced to exclude times where the signal is not present. The EU council recommendation from 1999 (25) which is based on ICNIRP 1998 (7) states that the limits are for instantaneous peak values. In the future, a more precise formulation would be desirable to avoid confusion.

Local exposure RL were not defined in ICNIRP 1998 (7), but it was stated that in addition to adhering to the whole-body RL, the BR on localized exposure are also not to be exceeded. This approach was unfortunate since it defied the idea that by complying with the RL, it can be assumed that the BR are not exceeded.

ICNIRP (1) takes a different approach and does not define additional limiting peak values for E- and H-fields. Starting from 400 MHz Table 7 defines frequency and duration dependent RL for local exposure in terms of U_inc_; please note the unusual use of RMS quantities to limit energy densities. This approach is somewhat similar to the limiting peak values from 1998 and follows the trend in numerical dosimetry. However, it is more difficult to apply in occupational risk assessment practice relying on *in-situ* exposure determination, since it does not directly limit E and H. Furthermore, it deals with local exposure and so there are no limiting peak values for whole-body exposure anymore. Below 400 MHz, ICNIRP states that “there is no brief-interval exposure level specified because, due to the large penetration depth, the total SA resulting from the 6-min local SAR average cannot increase temperature by more than the operational adverse health effect threshold (regardless of the particular pattern of pulses or brief exposures)” [(1), p. 490].

**Figure 1:**
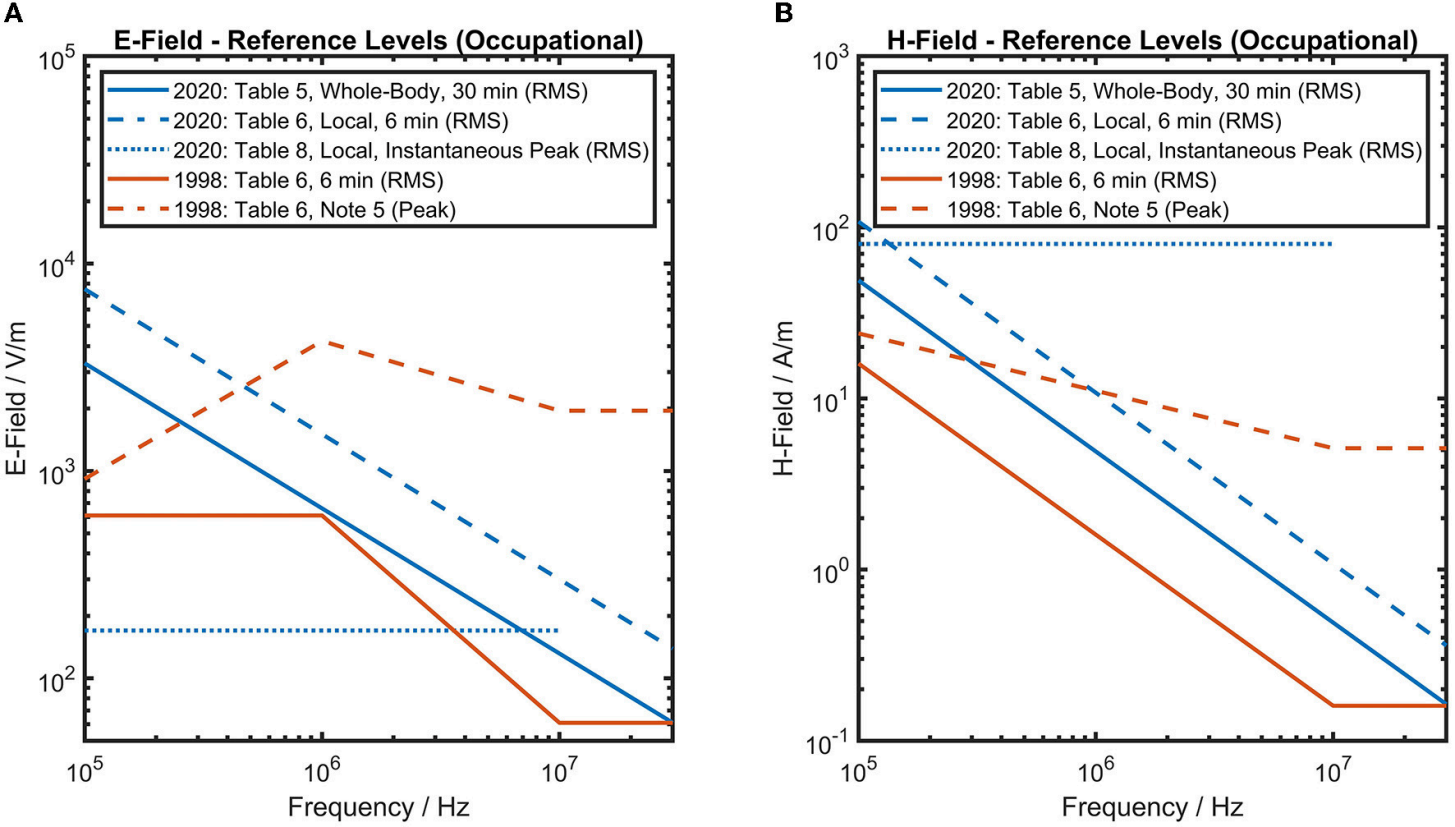
ICNIRP's 1998 and 2020 (1, 7) reference levels for occupational exposure are shown for E-field strength **(A)** and H-field strength **(B)** between 100 kHz and 30 MHz. Reference levels of ICNIRP 2020 (1) have increased. Since E- & H-field reference levels must both be considered, it is necessary to check compliance with six different values for frequencies between 100 kHz and 10 MHz—which is not ideal from a practical perspective.

Between 100 kHz and 30 MHz the RL were increased to “incorporate our [Note: ICNIRP's] improved knowledge” [(15), Section “Frequency Dependence of Reference Levels at Frequencies Below 30 MHz”]—please see Figure 1. As stated above, the peak value limitations of S_eq_ = 1,000 W/m^2^ have been dropped. ICNIRP 2020 (1) provides RL solely for peak instantaneous field strength measures in Table 8 (100 kHz−10 MHz), which are given as RMS values. The values are the same as the RL given in ICNIRP's 2010 guidelines (6) for protecting against nerve stimulation effects. Nerve stimulation effects depend on peak values. Hence it seems inappropriate to provide RMS quantities, which can be significantly smaller than peak values divided by √2 for sinusoids which include breaks. Furthermore, an instantaneous peak value cannot (by definition) be described as an RMS quantity. For application in occupational risk assessment, such confusion is challenging and a more precise formulation would be desirable.

In spite of the long-lasting tradition of complying with several RL for the same frequency range, it can still be quite confusing if not explained properly to the targeted audience. Please refer to Figure 1 for an attempt of clarification. ICNIRP 2020 (1) states that up to 30 MHz all exposures are to be treated as near-field exposures, such that both E and H-field RL must be complied with simultaneously. In practical applications this often leads to the situation that the H-field RL is the lowest and defines the limiting value. This should be considered when applying (1) to future European OSH legislation, since otherwise one needs to figure out which of the six offered RL marks the most conservative one for a particular exposure scenario at a workplace. In summary, proper application of the RL in OSH risk assessment practice seems to have become more difficult in comparison to ICNIRP 1998 (7).

In their description of the RL concept, ICNIRP states that: “In situations where reference level quantities are associated with greater uncertainty, reference levels must be applied more conservatively” [(1), p. 494]. Although it is understandable what motivates this statement, at the same time it is rather unfortunate and vague. If RL cannot simply be applied but must be checked for appropriateness without providing criteria for how to check such appropriateness, it makes their application in OSH risk assessment difficult and raises concerns about their conservativeness.

### Accuracy of Basic Restrictions, Reference Levels, and Averaging Times

In OSH risk assessment practice, *in-situ* measurements at the workplace to determine RF EMF exposure is very common, whereas simulation and numerical determination to determine either RL or BR prove rather challenging for reasons like costs and required expertise.

Combined with other factors impacting on the determined exposure, e.g., fluctuation of the measured quantity due to measurement equipment or source parameters, it is difficult to handle very precisely expressed RL with two digit accuracy. It proves especially challenging to explain such precisely expressed quantities in relation to measurement uncertainty (please refer to Section “Uncertainty of RF-Measurement and Reduction Factors for deriving Reference Levels”) and fail-safe safety measures when presenting the results of the risk assessment to experts in many other areas outside EMF OSH risk assessment.

### Time- and Spatial Averaging

To better understand how the difference between ICNIRP's 1998 and 2020 guidelines (1, 7) will affect measurements in workplaces, an occupational example concerning plastic sealers will be useful: High exposure to RF EMF can occur when operating dielectric heaters such as plastic welding machines, RF sealers, or glue dryers. In order to measure the E- and H-field strength from a plastic sealer one has to first make sure that one is selecting the worst-case scenario. The leakage fields from the machine depends on many things like type of plastic to be welded, number of layers, electrode length etc., please see further (26, 27). Before starting to take measurements a careful go through of the process is needed to see how the operator works and how they are positioned with regard to the electrodes.

**Figure 2:**
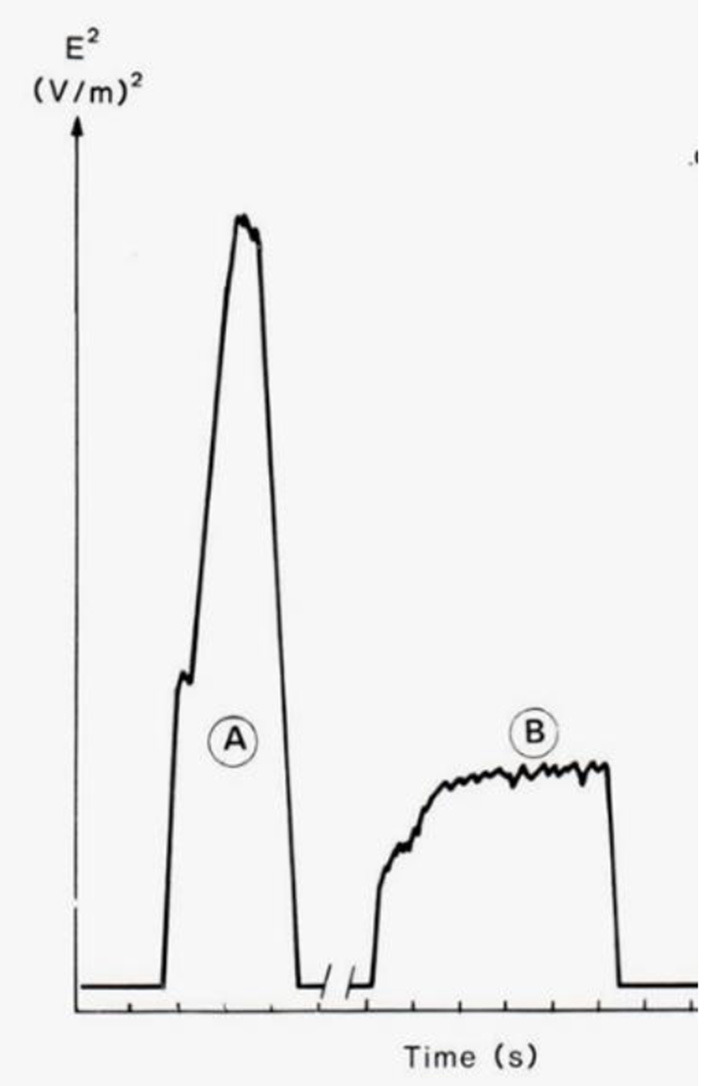
The time dependence of the squared electric field strength (E^2^) for two combinations of welding time and tuning, producing welding seams of the same quality (26). The area under the curves is approximately equal, which means that the same amount of energy is needed to produce the welding seams.

In addition, as can be seen in Figure 2, two different settings on the machine producing the same weld are shown. The leakage field is not constant during the welding time and no instrument available today integrates over the pulse, and therefore a peak hold approach is usually the answer. Working close to plastic sealers is within the reactive near field and measurements have to be taken both for the E- and H-field component.

There are some major differences between ICNIRP's 1998 and 2020 guidelines (1, 7) regarding time averaging as well as spatial averaging, which will affect the measurement procedures as well as the evaluation of the measured values. As stated previously, (1) includes reference values both as 6 min average as well as a 30 min average. Both are averages over the square values of E and H, but the volumes are different. According to Table 5 in ICNIRP 2020 (1), the 30 min period is averaged over the whole-body space and according to Table 6 the 6 min period is averaged over the relevant projected body space. Besides specifying the averaged surface area for calculation purposes of 1 and 4 cm^2^, (1) could be clearer about that and provide a quantitative rule to determine the actual body space projected by an EMF.

For OSH risk assessment in the case of plastic welding, the total time of the weld has to be recorded in order to make the time average over the 6 and 30 min time interval given in ICNIRP 2020 (1). Here the number of welds per 6 and 30 min period needs to be approximated and the total welding time added. Since the intensity of the leakage field depends on the setting of the machine, the worst case should be selected and the peak value is often used as a conservative measure. As an example, plastic welding machines are normally operating at 27 MHz. Thus, the relevant RL according to ICNIRP 2020 (1) is E_inc, 30min_ = 61 V/m, E_inc, 6min_ = 149 V/m. Based on previous measurements (28), a typical welding cycle could for instance be 10 welds, each 3 s long, performed each minute. At least for an older machine, a spatial average of the torso of E = 300 V/m and the whole-body of E = 60 V/m is possible. This means: here the effective welding time during an arbitrary 6 min period is 3 min, with an E_6minavg_ = 150 V/m and E_30minavg_ = 30 V/m. It is assumed that the stated welding pattern is repeated during at least 30 min; which might not be the case. In this example the spatial average of the torso has been used as local volume, but whether this is the relevant “projected body space” remains unsaid. Looking into the spatial variation of the field along the body axes, commonly the highest field strength is close to the waist since the electrodes often are in this position, and the field normally declines toward the head and feet. If the weld is performed manually, the hands and arms will experience relatively high exposure. What does this mean for the spatial averaging then? The whole-body average value could be rather straightforwardly calculated, if proper measurement has been done. But one needs to decide which volume/area constitutes the relevant projected body space and the choice of this might have large impact on the calculated local spatial average value. Concerning plastic welding it is worth noting that newer RF sealers are often well-shielded and grounded and that the workers exposure is often well-below the reference levels of ICNIRP 1998 and 2020 guidelines (1, 7).

Furthermore, it is not clear how to deal with the mean value from the spatial averaging for 30 min temporal averaging. Considering the spread in values—low values at foot, high at waist and low at head—the standard deviation will be large. How is this to be considered, when giving the value from the measurement to be compared with the reference values? If applying (1) to OSH risk assessment, more guidance to consider measurement uncertainty is strongly required.

An additional component in OSH risk assessment of RF sealers besides determining the E- and H-field strength is the determination of current induced in any limb and RF contact currents. ICNIRP 2020 (1) does not include limits for contact currents. The procedure for measuring the induced current is similar irrespectively of which guidelines (1, 7) are applied, except that (1) states that it only needs to be addressed when the worker is grounded. Introducing electrical insulated floor or gap will reduce the induced current considerably (29), but still needs to be assessed for efficiency of safety measures. For guidance on mitigation strategies (30) has published some useful mitigation measures for reducing the exposure of operators of RF sealers. These measures range from simple and costless to dedicated EMF shielding systems. Getting back to the application of ICNIRP 2020 (1) to OSH risk assessment, the provision of RL or at least recommendations for RF contact currents are required.

### Frequency Dependence of Reference Levels at Frequencies Below 30 MHz

Concerning the frequency dependence of the reference values, there are no major changes but the new guidelines have introduced a frequency dependence up to 30 MHz whereas in the earlier version and the directive the flat frequency response started at 10 MHz. Since a dielectric heater is operated at 13 or 27 MHz and operating frequencies are not perfectly stable during the welding process, this has to be taken account of. The implication of the novel frequency response curve, relevant e.g., for broadband probes, needs to be considered and incorporated in the measurement techniques used.

### Uncertainty of Dosimetric and Thermal Simulations in Basic Restriction Assessment at Frequencies Below 6 GHz

Dosimetric and thermophysiological simulation lay the basis for RF EMF exposure limits. Consequently, their trustworthiness and uncertainty of results require special attention. Major contributions to uncertainty are the following items:

1. Choice of the RF EMF source and exposure setup, e.g., position and posture of exposed workers.
2. Numerical errors due to spatial and temporal discretization.
3. Choice of tissue properties and their variability over the selected group of workers
  a. density, heat capacity, heat transfer rate, thermal conductivity
  b. dielectric properties
4. Modeling of blood flow and its dynamics (thermoregulation), and its variability over the selected group of workers.
5. Impact of environment such as ambient temperature and relative humidity, clothing.
6. Geometric size & shape of tissue
  a. basic anatomic models
  b. variation of size and weight over the selected group of workers

Although the first item clearly must be well-reflected for any occupational exposure assessment, it is acknowledged that RF EMF exposure limits are usually derived by simulation from the expected worst case setup. Computational errors due to the numerical solver algorithm, including stair-casing errors with state-of-the art (i.e., sub-mm) discretization of the geometrical setup, reach usually to not more than below 10% [1 s, please refer to (31), p. 53, Table 12, excluding tissue related uncertainty or (32)] and are hence only moderate.

On the other hand, uncertainty of simulation parameters such as tissue properties may be somewhat larger. In fact, many simulations use the same source of dielectric properties of tissue (33) which might obscure possible differences between simulations and consequently possible uncertainty. In fact, (34) refers to that as research gaps in computational dosimetry. As a lower bound of uncertainty, (33) the original statement of about 15% must be recalled.

Finally, the variation of body size, again over the selected group of workers, has undoubtedly the largest impact on uncertainty considerations. When using reference models for dosimetric simulations, the overall uncertainty should always be known, specified, and compared to the reduction factors of the BR. After all, the latter were explicitly derived to guarantee safe and healthy working conditions for all workers (e.g., female and male, tall and short), so that trust in the guidelines (1) is not undermined.

ICNIRP 2020 (1) is aware of some very specific exposure scenarios, in which exposure at the RL potentially leads to exceeding the BR: “small stature person (such as a 3-year-old child) to be extended (e.g., standing still and straight with arms above the head) for at least 30 min” [(1), p. 495–6]; also refer to Section “Problems With OAHET in Co-Exposure with harsh Work Environments”. Based on (35), the higher surface-mass ratio in small person results in a smaller temperature rise than would occur in a person of a larger stature. In any case, the resulting temperature rise would be substantially smaller than 1°C. Acknowledging ICNIRP's argumentation, from an occupational perspective the question does arise: How to deal with the resulting uncertainty reflecting e.g., the variability of workers and other EMF relevant individual characteristics (i.e., tattoos, piercings, medication etc.), their working postures, or work environment and use of tools?

**Figure 3:**
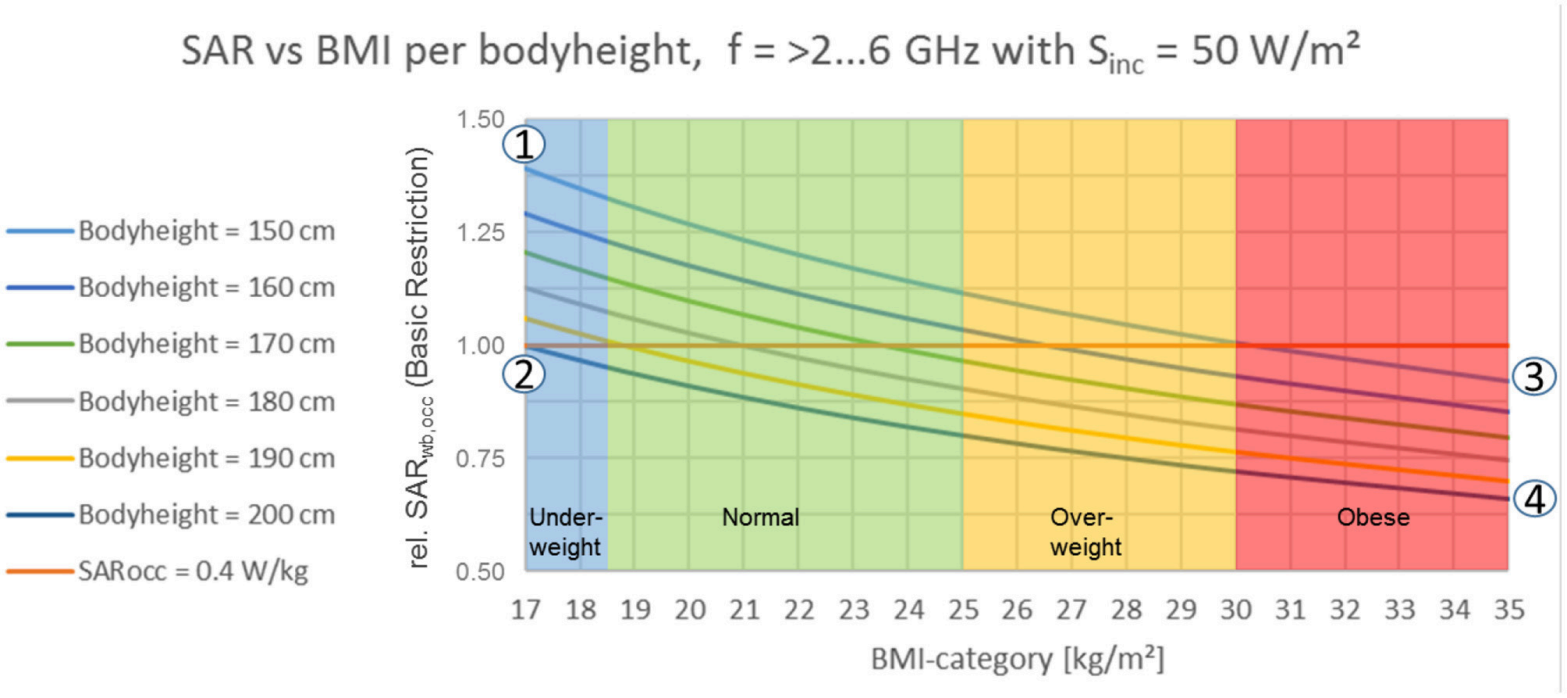
Whole-body: effect of body height on specific absorption rate in relation to body-mass-index category for frequencies >2–6 GHz, T_avg_. = 30 min [according to ICNIRP 2020 (1) Table 5].

**Figure 4:**
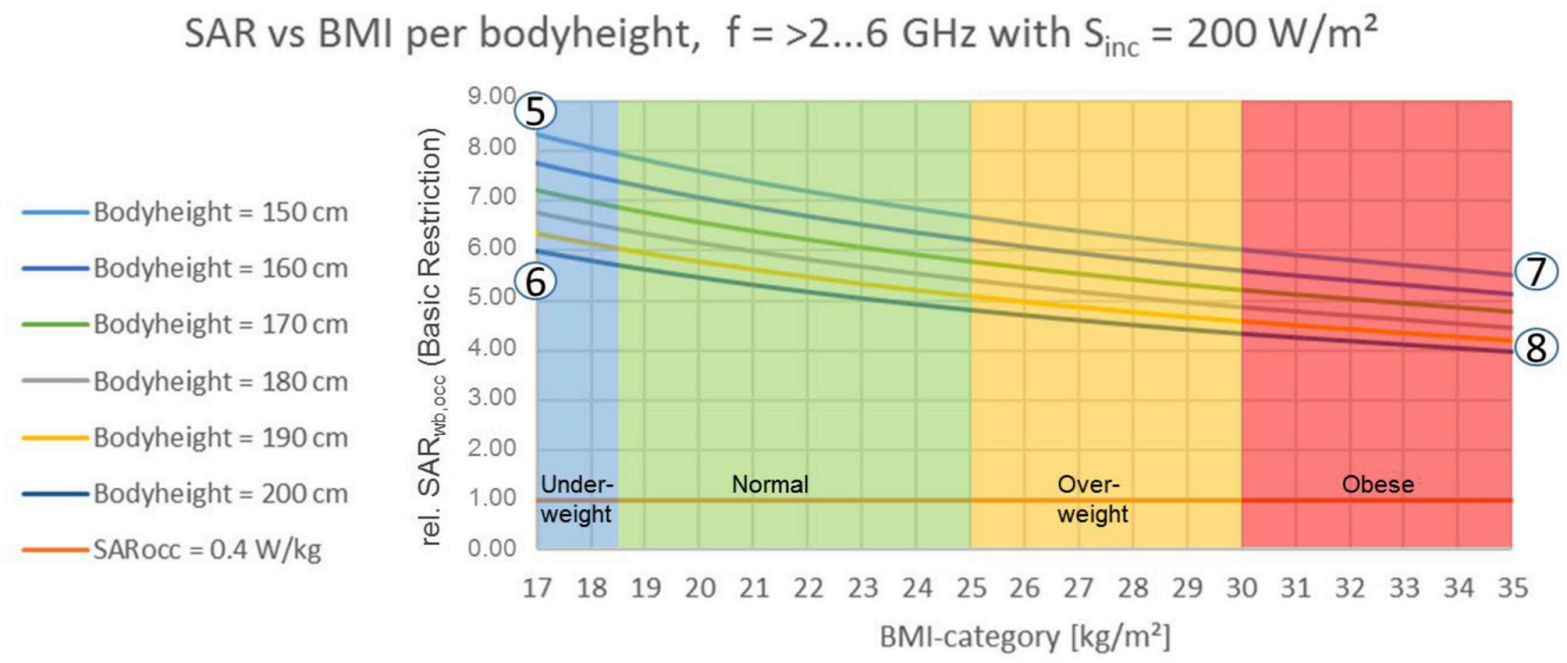
Unintended exposure of whole-body at local exposure levels, e.g., due to accident (with Body Surface Exposure Ratio c_pwbs_. = 1/2): Effect of body height on Specific Absorption Rate in relation to Body-Mass-Index Category for frequencies >2–6 GHz, T_avg_. = 6 min [according to ICNIRP 2020 (1) Table 6].


To visualize the uncertainty due to the variability of workers for OSH risk assessment, Figures 3, 4 display the effect of body height on the SAR level [as BR, Table 2 (1)] depending on body-mass-index category for EMF exposures of S_inc, wb_ = 50 W/m^2^ [as RL, Table 5 (1)] in the frequency range of 2 GHz < f ≤ 6 GHz.^3^ In occupational risk assessment, best coupling conditions are usually assumed to account for worst-case conditions. Hence the reflections coefficient is assumed to be 0, resulting in S_inc_ = S_ab_. Furthermore, S_inc_ is applied as RL, accessible via direct measurement at the workplace. Such exposure situations can be found e.g., in industry to dry and glue wooden materials or metal coated plastic materials, pest control with wooden materials, or climbing antenna sites. To quantify the resulting variability and hence uncertainty of BR and RL, the SAR level is calculated for a conservatively estimated projected whole body space of 1/3 (meaning only 1/3 of the whole-body surface is exposed); using equation 1 to 3 according to (36).

(1) $$SAR_{wba}=\frac{S_{inc}\cdot A_{\exp}}{m}$$

Equation 1, with Specific Absorption Rate (SAR in W/kg), incident Power Density (S_inc_ in W/m^2^), exposed Body Surface (A_exp_ in m^2^), Body Mass (m in kg)


(2) $$A_{\exp}=A_{wb}\cdot c_{pwbs}$$

Equation 2, with whole-body Body Surface (A_wb_ in m^2^), projected whole body space (conservatively estimated to be 1/3, c_pwbs_)


(3) $$A_{wb}=7.184\cdot 10^{-\;3}\cdot h^{0.725}\cdot m^{0.425}$$

Equation 3, with body height (h in m).

Referring to Figure 3 it should be noted that for exposures above 30 min averaging time according to ICNIRP [(1), Table 5] an elevation for BMI-normal and underweight workers can be observed, independently of working postures.

Figures 3, 4 and Table 1 show, within the underlying simple but essential model, the significant variation of wbaSAR over BMI per unit body height. The population data considered for calculation is based on (37), with:

- body height: 5th percentile of 148 cm (Japanese women) to 95th percentile of 196 cm (Dutch man) covering a variety of 48 cm and ±14% around the average.
- body weight: 5th percentile of 43 kg (Japanese women) to 95th percentile of 117 kg (Dutch man) covering a variety of 74 kg and ±46% around the average.

**Table 1:** Numerical values for reference points 1–4 according to Figures 3 and 5–8 according to Figure 2 (based on 33).

**Point#**	**Bodyheight (cm)**	**Bodyweight (kg)**	**SAR_**wb, occ**_, absolute (W/kg)**	**SAR_**wb, occ**_, relative**
1	150	38.0	0.56	1.40
2	200	78.8	0.37	0.93
3	150	68.0	0.40	1.00
4	200	140.0	0.26	0.65
5	150	38.0	3.34	8.35
6	200	78.8	2.21	5.53
7	150	68.0	2.40	6.00
8	200	140.0	1.59	3.98
5th percentile^*^	148	43.0	0.52	1.29
95th percentile^**^	196	117.0	0.30	0.74

* *BMI = 19.6 kg/m^2^,*

** *BMI = 30.5 kg/m^2^*.

Considering the evolution of anthropometric data as populations become taller and heavier (38, 39), the data published in 2013 adds more conservativeness to the presented results. Results from short, underweight persons (Figure 3 pt. 1, Figure 4 pt. 5) to tall, obese persons (Figure 3 pt. 4, Figure 4 pt. 8) differ about ±37% around the result for a “standard” person of 175 cm height. A workers' population within a range of 150–190 cm body height and a corresponding body weight of <66.6 ± 1.0 kg (for all BMI categories) is very likely to experience an EMF-related thermal load at BR level of SAR_wba_ = 0.4 W/kg or higher. Anatomical models are expected to show similar results by trend. For all dosimetric simulations that do not explicitly employ some worst case over the population (i.e., to model short, lightweight persons), these variations should be considered as uncertainty in view of the applicability to the overall population of workers. The statistical data of anatomical models may serve as a basis of a proper statistical interpretation of wbaSAR's and whole body RLs' variability with BMI and body height for workers. In conclusion, even with this simple model, we observe differences of a factor of about 2 between different body sizes and weight; not only limited to a 3-year-old child. ICNIRP's remark that some exposure scenarios at RL could potentially result in exceeding BR [(1), p. 495] may be an understatement for occupational exposure settings.

Finally, in OSH, selected unintended exposure situations with whole-body exposure at the level of the BR for local exposure ought to be considered for risk mitigation, e.g., accidents, rescue, or unintended use due to lack of training or dysfunctional mitigation measures. For risk assessment in this case, worst case exposure is assumed with a projected whole body space with a conservative c_pwbs_ = 1/2 at maximum local exposure of S_inc, local_ = 200 W/m^2^ (Table 6) in the frequency range of 2 GHz < f ≤ 6 GHz, by analogy with Figure 3. Such exposure situation could be applicable to workplaces, where maximum applicable exposure levels are determined by Table 6; with real exposure levels could certainly be much higher. According to equation 1–3, Figure 4 shows the resulting local SAR levels to be elevated (relatively to wbaSAR) by a factor of 4 for tall and obese workers (Figure 4 pt. 8) and a factor of 8 for small and underweight workers (Figure 4 pt. 5). From an OSH perspective it is only somewhat comforting that the assumption of applying local RL to the whole body space, which may elevate RF EMF exposure by a worst case factor of 8.34 as discussed above, does not exceed the whole-body reduction factor of 10. To be precise, the safety comfort for such unintended exposure situations diminishes to a factor of only (wbaSAR_OAHET_ = 4.0 W/kg)/(8.34 × wbaSAR_RB_ = 0.4 W/kg) = 1.2. Consequently, it would be desirable if future refinements of ICNIRP guidelines would address the issue of reduction factors adequately and would elaborate on the newly introduced local RL. Such knowledge would enable a purpose driven deduction of safety measures to guarantee safe and healthy working conditions.

### Uncertainty of RF-Measurement and Reduction Factors for Deriving Reference Levels

Article 4 paragraph 3 of the EMF directive states: “If compliance with the exposure limit values cannot be reliably determined on the basis of readily accessible information, the assessment of the exposure shall be carried out on the basis of measurements or calculations. In such a case, the assessment shall take into account uncertainties concerning the measurements or calculations, such as numerical errors, source modeling, phantom geometry and the electrical properties of tissues and materials, determined in accordance with relevant good practice” (4).

**Figure 5:**
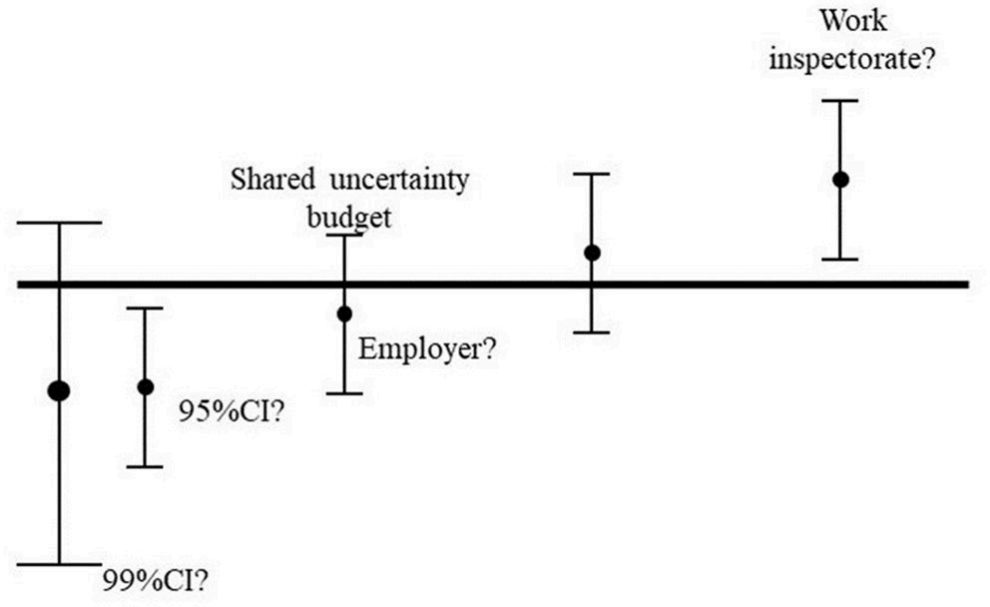
Example of different approaches in uncertainty evaluations depending on the purpose. The error bar visualize a strict legal perspective, the error bar to the left [95% confidence limit (CI)] could be seen from an employee's perspective—strictly below the limit; whereas the one to the right could be the view of the work inspectorate to see that the company legally are above the limit.

Figure 5 illustrates the problem of taking account of the uncertainty in measurement. The horizontal line represents a limit value to comply with, e.g., the action level of EMF (Directive 2013/35/EU) (4) or the E- or H-field RL of ICNIRP 2020 (1). The dot represents the mean value of the measurements with an instrument suitable for the situation. Now we add the uncertainties to the measurements, including both instrumental errors (non-isotropicity in the probe, non-linearity etc.) and calibration error, and the standard deviation of the measured values. Depending on the selection of 95 or 99% confidence interval, we get a different size of the error bars. If we go with the 95% confidence interval then we also have to be aware if we are doing the measurement for the employer or for the work inspectorate. The conservative approach then says that the measured value including the 95% confidence interval should stay below the limit value in order to show compliance. However, if the measurements are done by the work inspectorate then it works the other way, i.e., the whole 95% confidence interval should exceed the limit to infer that the workplace is not compliant.

In case a shared uncertainty budget is used, it is sufficient that the mean values are below or above the line to show compliance. This approach is often used when the error bars are small, but in case of RF EMF this is certainly not the case. Realistic examples of uncertainty calculations in EN 50413 (40) provide expanded uncertainty budgets to be considered in the range of 40 %.

**Figure 6:**
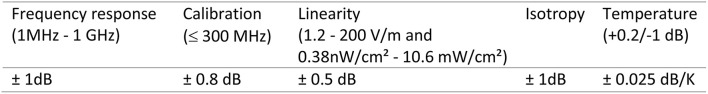
Example of uncertainties as given by narda sts for one of their electric field probes, 100 kHz−3 GHz [(37), p. 2].

Looking at one of the most common instrument used for measuring RF EMF we can see that the manufacturer specifies: Instrument error: ±1 dB frequency response, ± 1 dB for lack of isotropicity, ±1 dB from calibration; please refer to Figure 6 (41). In addition to uncertainty related to measurement equipment, the uncertainty of individual measurements need to be accounted for. Taking all of this into account it is hard to see how RF measurements in industrial settings could be done without including at least ±3 dB to the measured value when comparing with the action levels of EMF (Directive 2013/35/EU) (4).

When applying (1) to OSH risk assessment practice it is very important to clearly understand that there are no additional reduction factors applied when deriving RLs relevant to local and wbaSAR below 30 MHz [(1), p. 511]. This leads only to a small safety margin between RL and BR; which is comprehensible for computational exposure determination. However, as stated above, in practical OSH exposure determination *in-situ* measurements are the preferred means of choice. Consequently, an additive approach to account for measurement uncertainty is the only means to guarantee for safe and healthy work places, please refer to equation 4.


(4) exp+U≤ RL

Equation 4, with determined exposure (exp), expanded uncertainty (U), and reference level (RL)

At last just as a note, an indirect purpose of uncertainty assessment highlights its value: namely comparability of exposure levels between two risk assessments, industries at large, EU member states, or even the quality of risk assessment services.

## Open Questions in Practical Application and Research GAPS

### Spatial Averaging of Basic Restrictions and Local Reference Levels

Concerning practical applicability, the introduction of novel BR for brief local exposure, i.e., one that is shorter than 6 min, and novel RL for the two cases of “local” exposure (longer and shorter than 6 min) should be mentioned. Regarding a discussion about averaging time intervals, please refer to Section “Local Reference Levels for Exposure Durations of <6 Min”.

Apparently, specifications for local and brief exposure (100 kHz−6 GHz) is justified in ICNIRP's rationale by only a single reference [(42) as cited in ICNIRP 2020 (1)], from which the RL are directly derived. Spatial averaging of measurement values for comparison with local RL as of [(1), Tables 6, 7] is an open question for risk assessments at workplaces. Spatial averaging is specified by ICNIRP 2020 (1) in selected footnotes: notes 6–7 of Table 6 and notes 5–7 of Table 7, respectively. Besides volume and area metrics (namely “projected body space” and “projected body surface space”), ICNIRP 2020 (1) distinguished between “projected body space” and “projected whole-body space” for volume metrics (applicable for f <6 GHz). Practically speaking, (1) did not elaborate on spatial averaging criteria to show compliance, nor on compliance requirements in relation to what physical quantities to be used in near and far field zones. ICNIRP (1) rather describes their applicability quite generally as averages drawn over smaller body regions. In any case, in occupational exposure assessment one would almost always apply RL instead of BR, and such spatially distinctions between local and whole body averaging (coming with different temporal averaging as well) are quite difficult to apply in practical terms. With all that, when applying (1) to OSH risk assessment practice, selected open questions should be addressed, as in the corresponding rationale (1) is so far relatively silent on the following aspects:

- How were those RL chosen, particularly in the frequency range between 400 MHz and 6 GHz?
- What does the term “local” mean in the frequency range between 400 MHz and 6 GHz?
- Is spatial averaging for local exposure related to the spatial constraints of local BR, meaning limbs, head, and torso, or even tissue categories?
- Do the terms “projected body space” and “projected whole-body space” acknowledge that the EMF-source (e.g., its size, position in relation to a worker, aperture) propagates its EMF to a worker in terms of the incident spatial characteristics?
- It remains unclear how large this “local” and “projected space” could be:
  ° Should it compare to corresponding body parts such as the whole limb or the torso?
  ° Is it applied to sub-regions with those body parts, such as a hand holding a device?

Independently of answers to those questions, when applying (1) to OSH risk assessment practice it is necessary to prevent averaging out critical exposures by incompletely defining spatial averaging methods.

### Local Reference Levels for Exposure Durations of Shorter Than 6 Min

One of the two sets of “local” RL introduces specific values for brief exposures (shorter than 6 min) such as RF EMF pulses. With three sets of RL to be possibly applied, novel layers of complexity become possible in any occupational exposure assessment. As a result, we see the risk of diverging assessment results, using one or the other set of RLs, making them hard to compare and hard to understand for the average safety officer in industry.

The lack of limiting brief exposures by peak values is in contrast to both (2, 7). Both demanded a ceiling limit of a factor 1,000 for the peak power density averaged over the pulse was demanded. Any implementation into OSH-legislation must be aware of this novel aspect, and that thus limits may (by design) protect from thermal effects only, leaving other indirect effects of short pulses without limits to comply with. Such indirect effects, like microwave hearing, may still be distracting to workers. Especially the sensory effect of microwave hearing (mwh) may add auditory workload and therefore impedes the operation of stress- and strain- optimized workplaces. To quantify the situation based on (1 and 7), the following example is provided assuming a duty-on cycle of 1 s of an arbitrary EMF-source operating in the frequency range between 0.3 and 6 GHz: SA_local_ = 0.36 kJ/kg = 360 J/kg (according to 1, Table 3) compared to SA_mwh_ = 10 mJ/kg = 0.01 J/kg (according to 7, Table A2). Given appropriate exposure characteristics for the mhw-effect to possibly occur, it becomes very probable to really occur when exceeding the threshold for sensory effects (7) by a factor of 36,000.

### Guidelines on Workers at Particular Risk

Thresholds for the safety of workers at particular risk are outside the scope of (1). This is comprehensible, given the complexity of implants and underlying diagnoses. Anyhow, it is required to be regarded in OSH risk assessment (7, article 4 paragraph 5, number d). Hence, the following section aims to provide information rather than comparing (1 and 4).

A growing number of workers in the European Union wear Active or Passive Implanted Medical Devices (AIMD, PIMD) (43), which are prone to be influenced by RF EMF. ICNIRP (1) acknowledges that RF EMF can indirectly cause harm by unintentionally interfering with AIMD or interacting with conductive implants, but considers such exposures outside the scope of its guidelines [(1), p. 483], similar to ICNIRP 1998 (7). For OSH, where (individual) risk assessment for workers at particular risk is mandatory, other adequate guidelines are thus needed.

PIMD are comprised of any implantable medical device to replace or support impaired or lost body parts and their functionality, e.g., prostheses, stabilizers, stents, nails, screws, clips, artificial cardiac valves, or cranial plates. Besides those, metallic body jewelery and metallic pigments of tattoo ink may be affected by RF EMF. AIMD possess a source of energy and monitor, support, and/or replace impaired or lost body functions, like pacemakers, defibrillators, cochlear implants, or insulin pumps. At frequencies within the range of RF EMF, AIMD usually act like PIMD, meaning that the device and/ or its parts may distort EMF resulting in an altered or perturbed internal EMF. As a result, local field strengths may be increased leading to an increased SAR, i.e., rate of energy deposition, potentially harmful to surrounding tissues.

OSH risk assessment for workers at particular risk quickly becomes complex, often requiring individual case by case assessments to prevent workers at particular risk from unemployment and their employers from knowledge drain. Since (1) does not provide BR or RL for implant workers with AIMD or PIMD, it should be ensured when applying (1) to OSH risk assessment practice that such special guidance along with standardized exposure assessment procedures, and information to workers at particular risk are additionally provided to guarantee safe working conditions for workers at particular risk.

### Overall Uncertainty of Dosimetry Results Over the Group of Workers

To our knowledge, hardly any thorough analysis of the overall uncertainty of dosimetry results, over the population of workers, is available. Two of the presumably most important contributions would be, firstly, data of dielectric properties, where more research supporting the “Gabriel dataset” (31) primarily used until now is pending (34). These datasets were largely derived from measurements on excised and post mortem tissue up to 20 GHz, which might affect water content and thus dielectric parameters, as well as their applicability above 20 GHz. While reasoning on uncertainty is particularly relevant for millimeter wave exposure of superficial tissue such as skin, it might be extended to exposure scenarios involving contact with objects (such as those in a work environment), and in principle applies to dosimetry in general. Secondly, data on the impact of the statistical distribution of body sizes and weights would make the derivation of BR more transparent. An example of good practice are the low-frequency EMF limits by Reilly (44), which provide such data. Summing up all such contributions, the overall uncertainty of dosimetric simulations then must be put in the context of the reduction factors that link worst case effect thresholds (or OAHET) to the BR of RF EMF to guarantee safe and healthy working conditions under all circumstances.

Although (1) refers to some individual uncertainty contributions such as procedure or algorithm and skin conditions (dry or wet) with modest uncertainty, we are not aware of a state-of-the-art overall uncertainty analysis.

### Harsh (Work) Environments

More research is needed on the thermal effects in harsh environments and high ambient temperature (22). This would help to establish the validity of ICNIRP 2020 (1) also under harsh workplace conditions. Individual analysis for “verifying their body core temperature during work” as advised by ICNIRP 2020 [(1), p 500–501], is not feasible for risk assessments for workers with significant heat exposure from other sources. Usually, the tools and knowledge are either unlikely to be available to employers and workers or difficult to administer during day-to-day work routines.

### Exposure of Body-Regions, Partial Body Exposure (pbaSAR), and Local “Hot Spot” Effects

Most research so far focussed on either whole-body exposure, and its relevant quantity wbaSAR, and local “hot spots,” particularly in the region of the head (and e.g., narrow regions such as wrists). For those “hot spots,” the psSAR10g is identified as a relevant quantity. In view of

- occupational settings such as manual work near an RF sealer, and/or
- the novel RL of ICNIRP 2020 (1) for “local” exposure, it would be worthwhile to study thermal effects on larger body parts such as the limbs.

According to ICNIRP 2020 (1), the whole region of the limbs seems to be allowed to take psSAR10g = 20 W/kg and consequently +2.5°C, provided that the wbaSAR = 0.4 W/kg is met. We speculate, that in the aforementioned work situations, the introduction of a pbaSAR value could help to limit intense warming over larger body regions that would only be compensated by the rest of the body and its limits on body core temperature.

### Intense RF-Exposure of Humans

There is a remarkable lack of human *in vivo* RF-Exposure studies that would cover the full range of all occupational BR and ideally record also other endpoints than those directly associated with thermal effects. Almost all dosimetric studies and reviews of thermal effects in the sub-6 GHz range refer to—and for validation rely on—the classical studies of Adair [see e.g., (23)] from about 20 years ago. In those studies, exposure focused on wbaSAR and duration was below 1 h, which thus marks the level of validation reached by *in vivo* experiments, although BR are applicable to exposure of any duration. Good exposure studies near the occupational BR, and with relevant duration with respect to working hours, simply do not exist to our knowledge; please refer to Section “Introduction”.

### Exposure Duration in Occupational Exposure Settings

To reduce the uncertainty of extrapolating from laboratory animal exposure studies at mobile communication frequencies with exposure levels below or at general public limits, *in situ* study designs using realistic work place exposure scenarios are highly encouraged. This could improve scientific evidence (e.g., as starting point for epidemiological analyses) around occupational exposure durations during working hours over decades at maximum permissible occupational exposure levels along with other typical occupational criteria such as worker's anatomy, working positions, physical activity, harsh environments, tools, occupational EMF sources with frequencies far below the GHz range, and occupational field characteristics, such as frequency or pulsation.

Furthermore, there is a lack of knowledge about possible interaction mechanisms, biological reset time (e.g., time of biological system to recover from RF EMF induced damages, duration of repair mechanisms), temporal characteristics, and other dose related criteria (45).

## Concluding Remarks

We highly acknowledge the effort and scientific quality that is shown by ICNIRP 2020 (1). This review aims to foster a constructive dialogue aimed at future improvements. Based on previous experiences with the implementation of ICNIRP guidelines in OSH risk assessment, it became necessary to reflect the 2020 ICNIRP RF EMF guidelines (1) in the light of OSH risk assessment practice. Occupational exposure settings differ significantly to those of the general public, with the latter ones focussing more or less on the application of mobile communication with limited frequency ranges and exposure levels. In contrast, occupational exposure is permitted at levels 5 times larger than the general public, across the whole frequency range from 100 kHz to 300 GHz with unlimited exposure durations. ICNIRP did not provide practical details relevant to OSH exposure assessment and occupational exposure durations during working hours over decades as well as its effects. Some of these details could be addressed in an updated version of the non-binding guide for the EMF Directive (8, 9) or could be provided in relevant technical standards. Other, more fundamental choices that are necessary to provide legal clarity could be addressed in a possible revision of the EMF directive.

The removal of non-adverse health effects, e.g., the microwave hearing effect, is questionable for OSH purposes. In consequence, a solid anchor in OSH legislation is required to provide a reliable basis to evaluate distracting effects, even if they are not adverse to health, in OSH risk assessments.

In addition, several open questions regarding ICNIRP's (1) practical implementation were identified and discussed in the present review. It is acknowledged that ICNIRP itself has no intention nor obligation to consider the practical application of its guidelines in OSH tasks, such as workplace risk assessment. As challenging as it might be, such considerations are clearly needed for a reliable OSH risk assessment practice. Those considerations are yet to be developed, along with appropriate measurement devices. To facilitate a holistic understanding and application of ICNIRP 2020 (1), a set of BR and RL which are easy to comprehend is desirable for OSH practitioners. Furthermore, any doubt on conservativeness, for all possible occupational scenarios is disadvantageous, and should be avoided by an appropriate selection of BR and RL. Considering policy makers at a European and member states level, OSH EMF-legislation would benefit greatly from an adopted safety concept, comprising a comprehensible set BR and RL for all substantiated RF EMF effects, regardless of whether they are adverse to health or distracting.

## Author Contributions

PJ: introduction and concluding remarks. KH, RS, and JW: fundamentals of ICNIRP RF guidelines 2020: what is different for workplace assessment? CA, PJ, KS, and FS: description of ICNIRP's operational adverse health effects thresholds and characterization of deeper tissue. KH, PJ, KS, FS, and JW: practical aspects of compliance assessment. CA, KH, MIs, MIv, PJ, KS, TS, RS, and JW: open questions in practical application and research gaps. CA, KH, MIs, MIv, PJ, KS, TS, FS, RS, and JW: revision. All authors contributed to the article and approved the submitted version.

## Conflict of Interest

The authors declare that the research was conducted in the absence of any commercial or financial relationships that could be construed as a potential conflict of interest.

## Publisher's Note

All claims expressed in this article are solely those of the authors and do not necessarily represent those of their affiliated organizations, or those of the publisher, the editors and the reviewers. Any product that may be evaluated in this article, or claim that may be made by its manufacturer, is not guaranteed or endorsed by the publisher.

## Footnotes

^1^Frankfurt Agreement defines the cooperation between IEC and CENELEC concerning the development of new standards aiming to avoid duplication and to reduce preparation time, CENELEC supports the primacy of international standardization (~80% of CENELEC standards are identical or based on IEC publications, https://www.cencenelec.eu/about-cenelec/cenelec-and-iec-cooperation/, accessed January 13, 2022).

^2^OAHET +5°C/2 allows max. +2.5°C.

^3^For the frequency range between 2 GHz < f <6 GHz, S_inc_ as RL is applied to enable the comparison with the SAR (as BR), instead of the S_ab_ (BR) as applicable for frequencies above 6 GHz.
